# Smoking-Mediated miR-301a/IRF1 Axis Controlling Immunotherapy Response in Lung Squamous Cell Carcinoma Revealed by Bioinformatic Analysis

**DOI:** 10.3390/cancers16122208

**Published:** 2024-06-13

**Authors:** Alina M. Perevalova, Vladislav V. Kononchuk, Tatiana S. Kalinina, Vadim V. Kozlov, Lyudmila F. Gulyaeva, Vladimir O. Pustylnyak

**Affiliations:** 1 Zelman Institute for the Medicine and Psychology, Novosibirsk State University, Pirogova Street, 1, 630090 Novosibirsk, Russia; a.perw@yandex.ru (A.M.P.); gulyaeva@niimbb.ru (L.F.G.); 2 Federal Research Center of Fundamental and Translational Medicine, 630117 Novosibirsk, Russia; kononchuk@niimbb.ru (V.V.K.); kalinina@niimbb.ru (T.S.K.); vadimkozlov80@mail.ru (V.V.K.); 3 Novosibirsk Regional Oncology Center, 630108 Novosibirsk, Russia

**Keywords:** squamous cell lung cancer, smoking, miR-301a, IRF1, immunotherapy

## Abstract

**Simple Summary:**

The impact of smoking on the development of lung cancer is widely acknowledged; however, all of the molecular processes behind this effect, as well as their possible clinical applications, are still unclear. The current study aimed to identify and describe a potential smoking-related molecular mechanism in squamous cell lung cancer, with its possible connection to the immunotherapy response. By analyzing bioinformatic data from different databases and conducting experiments on clinical tumor samples, we demonstrated the connection between the microRNA mir-301a and the transcription factor IRF1, which demonstrated the capacity to affect many immune pathways in lung cancer and the efficacy of treatment with immune checkpoint inhibitors. These findings not only clarify certain molecular characteristics of squamous cell lung cancer but also open up new avenues for future immunotherapy research.

**Abstract:**

Smoking is an established risk factor for a variety of malignant tumors, the most well-known of which is lung cancer. Various molecular interactions are known to link tobacco smoke exposure to lung cancer, but new data are still emerging on the effects of smoking on lung cancer development, progression, and tumor response to therapy. In this study, we reveal in further detail the previously established association between smoking and hsa-mir-301a activity in lung squamous cell carcinoma, LUSC. Using different bioinformatic tools, we identified IRF1 as a key smoking-regulated target of hsa-mir-301a in LUSC. We further confirmed this relationship experimentally using clinical LUSC tissue samples and intact lung tissue samples. Thus, increased hsa-mir-301a levels, decreased IRF1 mRNA levels, and their negative correlation were shown in LUSC tumor samples. Additional bioinformatic investigation for potential pathways impacted by such a mechanism demonstrated IRF1’s multifaceted role in controlling the antitumor immune response in LUSC. IRF1 was then shown to affect tumor immune infiltration, the expression of immune checkpoint molecules, and the efficacy of immune checkpoint blockade therapy. As a result, here we suggest a smoking-regulated mir301a/IRF1 molecular axis that could modulate the antitumor immune response and immunotherapy efficacy in LUSC, opening up novel opportunities for future research.

## 1. Introduction

Lung cancer (LC) remains the most common cause of cancer-related deaths and the most common type of cancer diagnosed, according to the GLOBOCAN 2022 cancer incidence and mortality data [1]. Non-small-cell lung cancer, NSCLC, is believed to account for more than 85% of all lung malignancies, with the most frequent subtypes being lung adenocarcinoma (LUAD) and lung squamous cell carcinoma (LUSC). Smoking continues to be the primary risk factor for LC, having the highest relative risk score when compared to other risk factors. Moreover, LC incidence rates are often associated with differences in tobacco usage patterns between different geographic regions [2]. It has also been demonstrated that quitting smoking even shortly before receiving cancer treatment improves the prognosis for LC patients [3]. Compared to LUAD, LUSC shows a stronger association with smoking and lower patient survival rates [4,5]. Many potential molecular mechanisms that may be related to smoking in the initiation and progression of LC are still being investigated. Tobacco usage has been connected to a number of molecular and cellular pro-carcinogenic pathways, including ROS-induced DNA damage, DNA methylation, activation of various signaling pathways, epithelial–mesenchymal transition, remodeling of the tumor immune microenvironment, and many others [6,7,8,9,10,11]. Potential diagnostic and prognostic biomarkers, as well as novel therapeutic targets, are constantly being researched for the treatment of LC [12,13]. The short non-coding RNAs, or microRNAs, have gained a lot of interest in this view; numerous studies have investigated the role of microRNAs in the development of various types of malignant tumors, including LC. Multiple microRNAs are being studied as potential biomarkers for LC. For example, the expression levels of certain miRNAs were associated with specific histological tumor subtypes, patient prognosis, and response to treatment [14]. Several microRNAs are known to be involved in a number of carcinogenic pathways in LC [15]. There is also an extensive amount of research linking certain microRNAs to the smoking-dependent development of LC and other lung diseases [16,17]. Nevertheless, further research is needed to determine whether their potential molecular interactions and effects on LC development offer good prospects for potential future clinical applications.

Previously, we used bioinformatic data to demonstrate a significant increase in hsa-mir-301a levels in LUSC samples from the TCGA dataset of the UALCAN database. Moreover, it was observed that hsa-mir-301a levels in LUSC tumor tissues were significantly higher in patients with a positive smoking status [18]. Based on these findings, we then hypothesized that smoking could be a factor contributing to higher hsa-mir-301a levels in LUSC samples. Research on hsa-mir-301a’s involvement in the development of malignant tumors is ongoing; for example, previous studies have already shown its impact on pro-inflammatory conditions and tumor progression in lung and colon cancer [19]. However, all the details regarding the molecular interactions it is involved in, as well as its relationship with smoking (a major risk factor for LC), are still unclear. The aim of the current study was to investigate the previously discovered association between hsa-mir-301a and smoking in LUSC and to explore the possible interactions underlying it.

## 2. Materials and Methods

### 2.1. UALCAN Database Analysis

In order to analyze the mRNA and miRNA levels in LUSC tissues compared to those in normal lung tissues, TCGA data were accessed using the UALCAN database (http://ualcan.path.uab.edu/analysis.html, (accessed on 7 May 2024). Next, a subgroup analysis was performed using patient clinical data, which included smoking status for patients diagnosed with LUSC [20].

### 2.2. MirDB Database Analysis

The MirDB database (https://mirdb.org/, (accessed on 7 May 2024) was applied for hsa-mir-301a-3p target prediction. The predicted targets with target scores of 90 and above were obtained from the database for further analysis [21].

### 2.3. LinkedOmics Database Analysis

The LinkedOmics online platform [22] (http://www.linkedomics.org/, (accessed on 7 May 2024) was used to analyze the expression of genes negatively correlating with hsa-mir-301a-3p in the TCGA dataset of LUSC samples. The Spearman correlation coefficient was used for statistical analysis.

### 2.4. TargetScan Database Analysis

The TargetScan algorithm (https://www.targetscan.org/vert_80/, (accessed on 7 May 2024) was applied to investigate all potential sites of hsa-mir301a-3p and IRF1 mRNA interaction [23].

### 2.5. Metascape and Cancerhallmarks Databases Analysis

After testing the hypothesis on clinical samples, databases were used to identify potential pathways that could be influenced by the mir301a/IRF1 axis in LUSC. For pathway analysis, enrichment plots for target gene lists were obtained from the Metascape (https://metascape.org, (accessed on 7 May 2024) and Cancerhallmarks.com databases [24].

### 2.6. TFlink Database Analysis

The list of IRF1′s known target genes was obtained from the TFlink database (https://tflink.net/, (accessed on 7 May 2024), which provides information on target gene interactions, nucleotide sequences, and the genomic locations of transcription factor binding sites [25].

### 2.7. TIMER Database Analysis

The DiffExp module of the TIMER database [26] (https://cistrome.shinyapps.io/timer/, (accessed on 7 May 2024) was applied to examine the expression of the IRF1 gene in a pan-cancer view. The gene module was applied to visualize the correlation of IRF1 expression with tumor immune infiltration level in LUSC samples.

### 2.8. KMplot Database Analysis

The immunotherapy module of the KM plotter database (https://kmplot.com/analysis/, (accessed on 7 May 2024) was accessed to analyze the association of IRF1 expression status with immunotherapy response in LUSC patients [27].

### 2.9. Patients and Tissue Collection

Tissue samples from 56 LUSC patients who underwent complete surgical resection at Novosibirsk Regional Clinical Oncology Center in 2021–2022 were used in this study. Each patient’s samples included both tumor tissues and matched adjacent non-cancerous lung tissues. Patients who had received neoadjuvant therapy or were diagnosed with distant metastasis at the initial presentation were not included. Clinicopathological characteristics were collected during the enrollment period by review of medical records. Each patient’s diagnosis was confirmed through pathological examination. After resection, all tissue samples were immediately frozen in liquid nitrogen and stored at −80 °C. All 56 patients gave written informed consent during the treatment periods. The study was approved by the Ethics Committee of the Federal Research Center for Fundamental and Translational Medicine (Novosibirsk, Russia, protocol code #4/2021), in accordance with the Helsinki Declaration of 1975.

### 2.10. Total RNA Extraction and the Real-Time PCR

In order to measure microRNA and mRNA expression levels, we followed previously published protocols for isolating microRNA and total RNA, reverse transcription, and real-time PCR [28]. The TRIzol™ reagent (Invitrogen, Waltham, MA, USA) was used to isolate the total RNA, the concentration and purity of which were measured using the Agilent-8453 spectroscopy system (Agilent Technologies, Santa Clara, CA, USA). The integrity of the isolated RNA was assessed using agarose gel electrophoresis. The RT-M-MuLV-RH kit (BiolabMix, Moscow, Russia) was used for reverse transcription, and stem-loop RT-PCR was performed using reverse transcribed total RNA with specific stem-loop primers. Using the CFX96™ Detection System (Bio-Rad Laboratories, Hercules, CA, USA), Ct values were measured by real-time PCR using the HS-qPCR SYBR Blue (2×) kit (Biolabmix, Novosibirsk, Russia) for IRF1, 18S, and POLR2A mRNAs and the UDG HS-qPCR (2×) kit (Biolabmix, Novosibirsk, Russia) for hsa-mir-301a-3p, U44, and U48 snRNAs. The reverse transcription reaction and PCR were performed using specific oligonucleotide sequences (Supplementary Table S1). For each reaction, Ct values were normalized to the geometric means of the 18S and POLR2A mRNAs for IRF1, and the U44 and U48 snRNAs for miRNA.

### 2.11. Statistical Analysis

Due to the data’s non-normal distribution, non-parametric tests were used for analysis. The non-parametric two-tailed Mann–Whitney U-test was applied for statistical analysis using MedCalc v20.011 software. The correlations of the values were examined using a Spearman correlation analysis. A *p*-value of < 0.05 was considered statistically significant.

## 3. Results

### 3.1. The Main Smoking-Related Target of miR301a Is IRF1

**Figure 1:**
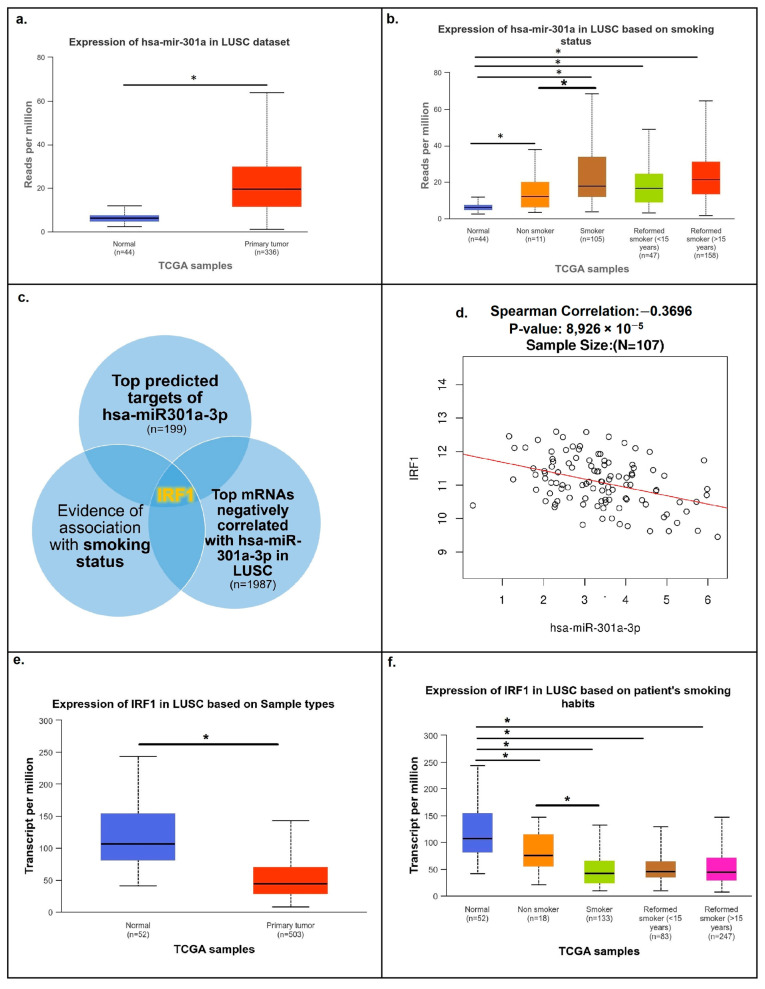
Analysis of major targets for the hsa-mir-301a-3p microRNA in LUSC. hsa-mir-301a expression in LUSC based on sample types (**a**) and patients’ smoking status (**b**) from the TCGA dataset of the UALCAN database. Levels of hsa-miR301a are elevated in tumor samples compared to normal lung tissue samples, and they are higher in samples from patients with a positive smoking status. (**c**) IRF1 mRNA was revealed as hsa-mir-301a’s only target with a high prediction target score (MirDB database), negatively correlated with hsa-mir-301a-3p in LUSC (LinkedOmics database), and associated with patients’ smoking status (UALCAN database). (**d**) Spearman correlation plot showing a significant negative correlation between hsa-miR301a-3p and IRF1 mRNA in LUSC samples (LinkedOmics database). (**e**,**f**) IRF1 mRNA levels are significantly reduced in LUSC tumor samples and in samples from patients with a positive smoking status (UALCAN database). * *p* < 0.01.

In order to investigate in more detail the previously identified increase in hsa-mir-301a levels in LUSC and its possible association with smoking (Figure 1a,b), we attempted to explore potential molecular interactions that might be involved. To assess possible smoking-related molecular interactions involving hsa-mir-301a-3p, we first investigated its major molecular targets. The list of top genes with the highest prediction target scores (scores of 90 or more) was compiled from the MirDB database and included 199 genes (Supplementary Table S2). Next, using the CPTAC LinkedOmics dataset, we compared this list to the list of top mRNAs that negatively correlated with hsa-mir-301a-3p in LUSC samples (mRNAs with Spearman correlation coefficients of −0.35 or lower were analyzed; 1987 gene names in total were included; Supplementary Table S3). Between the two lists, a total of 19 gene names were shared; however, only 1 of these 19 genes, IRF1, was found to be significantly associated with the patient’s smoking status, according to the UALCAN TCGA data (Figure 1c; Supplementary Table S4). IRF1 mRNA was then shown to be significantly downregulated in LUSC tumor samples, lower in smokers, and negatively correlated with cellular levels of hsa-mir-301a-3p (Figure 1d–f). Moreover, the TargetScan database also indicates that IRF1 mRNA has two highly conserved and two poorly conserved hsa-mir-301a-3p binding sites, illustrating their close relationship.

**Figure 2:**
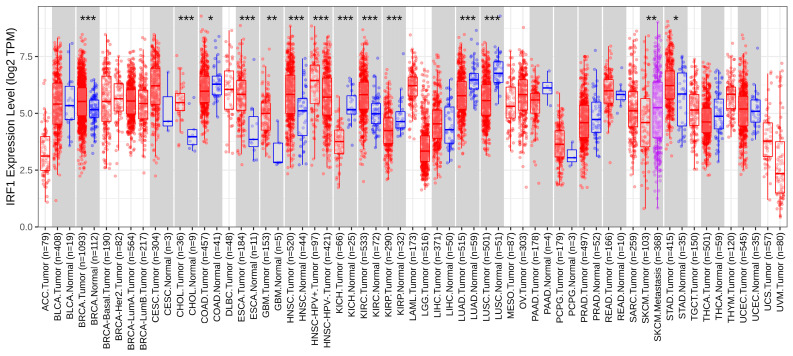
Pan-cancer IRF1 expression (TIMER database). IRF1 is significantly downregulated in both LUSC and LUAD non-small-cell lung cancer subtypes. * *p* < 0.05; ** *p* < 0.01; *** *p* < 0.001.

According to the TIMER database’s pan-cancer gene expression viewpoint, IRF1 was shown to be significantly downregulated in five human cancer types (including LUSC and LUAD lung cancers) and significantly upregulated in seven cancer types compared to normal samples (Figure 2).

### 3.2. Negative Correlation between miR301a and IRF1 Was Demonstrated in LUSC Clinical Samples

In order to validate these bioinformatic findings, we additionally performed RT-PCR experiments analyzing the cellular levels of hsa-mir-301a-3p and IRF1 mRNA in clinical LUSC tumor samples. After isolating total RNA and microRNA from clinical tissue samples, a reverse transcription reaction was conducted for each sample, followed by real-time PCR with specific oligonucleotide primers. For each of the 56 patients, samples included both malignant tumor tissues and corresponding adjacent non-cancerous lung tissues. Table 1 contains the clinicopathological characteristics of LUSC patients included in this study.

**Figure 3:**
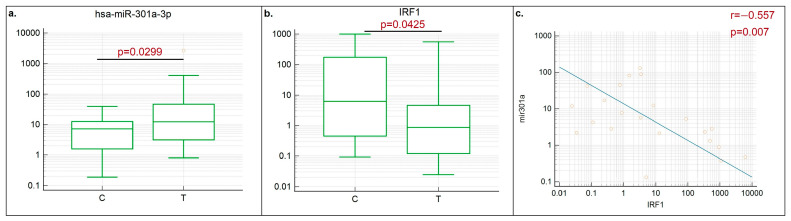
Hsa-mir-301a-3p (**a**) and IRF1 mRNA (**b**) levels in LUSC clinical tumor samples, with a Spearman correlation plot demonstrating a negative correlation between IRF1 mRNA and hsa-mir-301a-3p levels in LUSC tumor samples (**c**).

Consistent with previous bioinformatics research, the results demonstrated that hsa-mir-301a-3p was upregulated in tumor samples, while the IRF1 mRNA was downregulated (Figure 3a,b). A negative correlation between hsa-mir-301a-3p and IRF1 mRNA cellular levels was also demonstrated (Figure 3c). Notably, lung tissues from healthy individuals were used to normalize the expression data in the previously mentioned databases; however, our study showed comparable results when using adjacent non-cancerous tissues for normalization. Unfortunately, the low incidence of LUSC in individuals with a negative smoking status did not allow us to obtain a sufficient number of samples to form a distinct subgroup for comparison. Nonetheless, the findings observed in LUSC tumors in a subset of smokers are consistent with previously described bioinformatic data on the relationship between IRF1 and hsa-mir-301a-3p.

### 3.3. IRF1′s Function in LUSC

**Figure 4:**
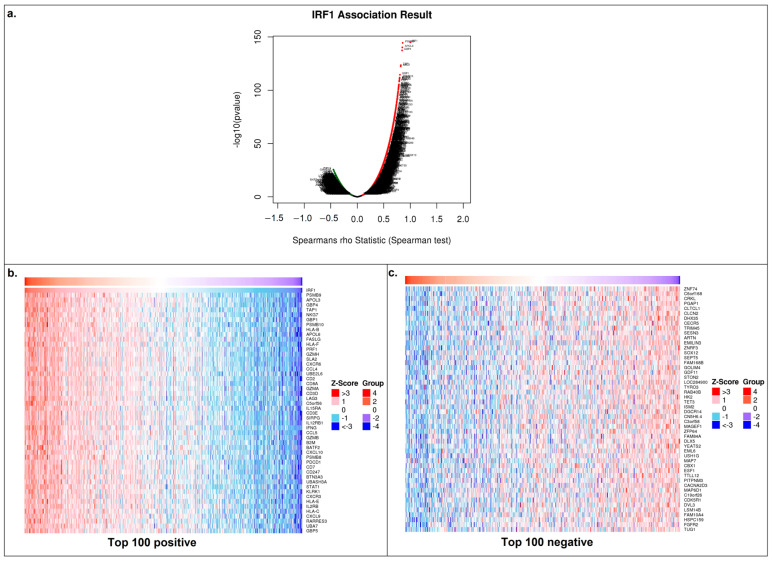
Genes correlating with IRF1 in LUSC. (**a**) Correlation plot for IRF1 mRNA from the TCGA dataset of the LinkedOmics database. (**b**,**c**) Heatmaps for genes of the top 100 positively and top 100 negatively correlated mRNAs.

After validating the hypothesis on clinical samples, we used databases to identify potential pathways that could be affected by the mir301a/IRF1 axis in LUSC. Our next step was to examine the list of genes that had the highest and lowest correlation coefficients with IRF1 in order to evaluate any potential effects that IRF1 may have on LUSC cells. TCGA data on mRNAs correlating with IRF1 mRNA in LUSC samples were collected from the LinkedOmics database (Figure 4a–c).

**Figure 5:**
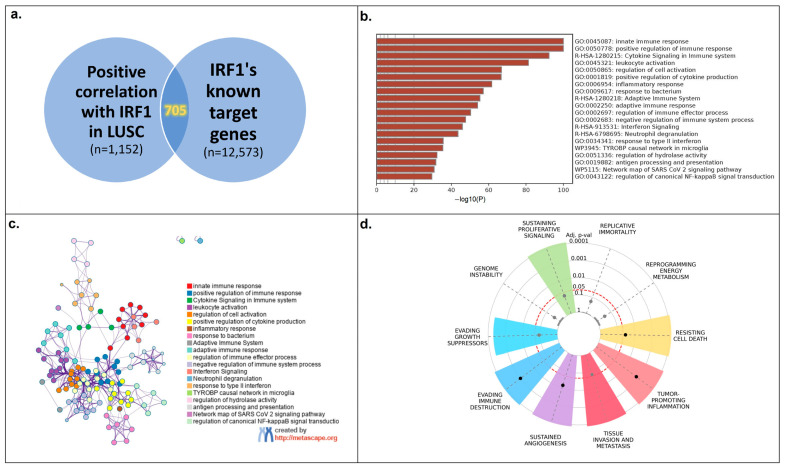
Pathway analysis for genes whose mRNAs correlated positively with IRF1 mRNA in LUSC. (**a**) Of all the IRF1 correlations detected, 705 genes were found to be its established transcriptional targets (LinkedOmics and TFlink databases). (**b**,**c**) Analysis of pathways induced by the resulting group of IRF1 targets. Most of the induced pathways were found to be associated with the immune response (Metascape database). (**d**) Cancer hallmarks plot showing the involvement of 7 common hallmarks (Cancerhallmarks.com database).

Positively correlated genes with a Spearman correlation coefficient of 0.35 or greater (a total of 1152 genes; Supplementary Table S5) were then compared to the TFlink database’s list of known IRF1 target genes (a total of 12,573 genes; Supplementary Table S6), yielding 705 genes that were shared by both groups (Figure 5a; Supplementary Table S7). To determine the main processes impacted by IRF1 in LUSC, this gene list was subsequently examined using the Metascape and Cancerhallmarks databases.

According to these data, the majority of the enriched processes are engaged in the control of diverse immune responses (Figure 5b,c), which is consistent with IRF1’s known functions in regulating antitumor immunity. Additionally, the hallmark enrichment plot demonstrated the involvement of seven common cancer hallmarks (Figure 5d). After repeating the same process with the top 100 negatively correlated genes, 60 of them were discovered as IRF1 target genes; however, no functional connections to cancer-related pathways were observed in the abovementioned databases.

### 3.4. IRF1 Controls the Antitumor Immune Response in LUSC

**Figure 6:**
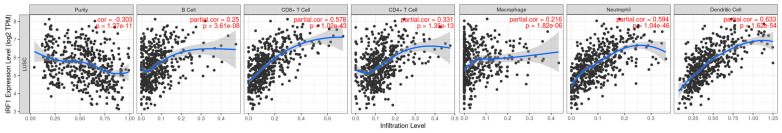
IRF1 correlations with tumor immune infiltration. *IRF1* expression correlated positively with the level of all analyzed immune cell types and negatively with tumor purity.

To evaluate whether IRF1 was involved in modulating the antitumor immune response in LUSC, we used the immune association module from the TIMER database. It clearly established a positive correlation between IRF1 expression level and the level of tumor immune infiltration in LUSC samples. Such a correlation was observed for B cells, CD8+ T cells, CD4+ T cells, macrophages, neutrophils and dendritic cells. A significant negative correlation between *IRF1* expression and tumor purity was also demonstrated (Figure 6).

**Figure 7:**
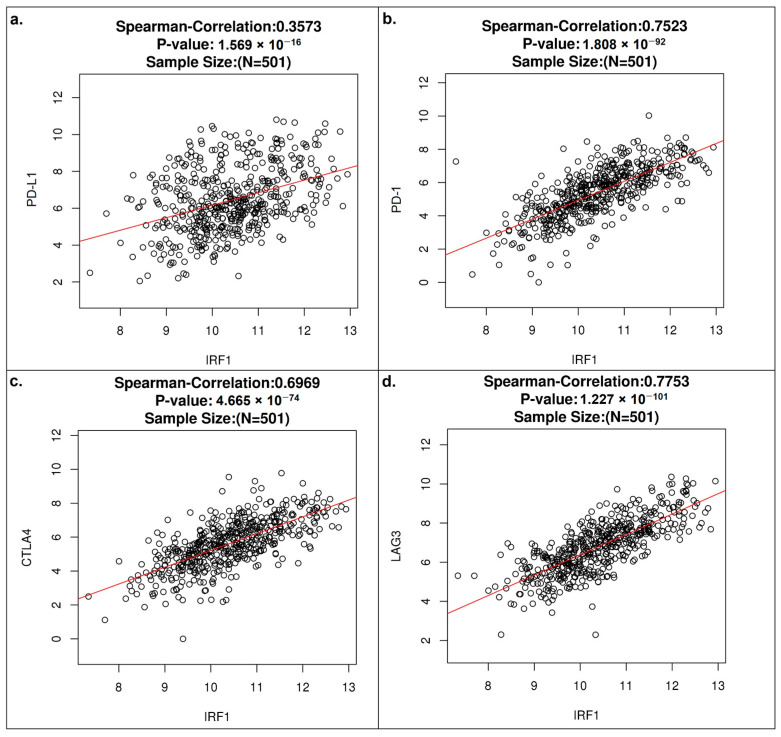
Positive correlation of IRF1 expression with immune checkpoint molecules in LUSC. Spearman correlation plots showing the correlation of IRF1 mRNA with mRNAs of (**a**) PD-L1, (**b**) PD-1, (**c**) CTLA-4, and (**d**) LAG-3 immune checkpoints (LinkedOmics database).

To obtain more details about IRF1’s immunomodulatory activity in LUSC, we investigated its relationships with the most typical immune checkpoint molecules. The TCGA-LUSC dataset from the LinkedOmics database showed a positive correlation of IRF1 mRNA with all PD-L1, PD-1, CTLA-4, and LAG-3 mRNAs (Figure 7a–d).

**Figure 8:**
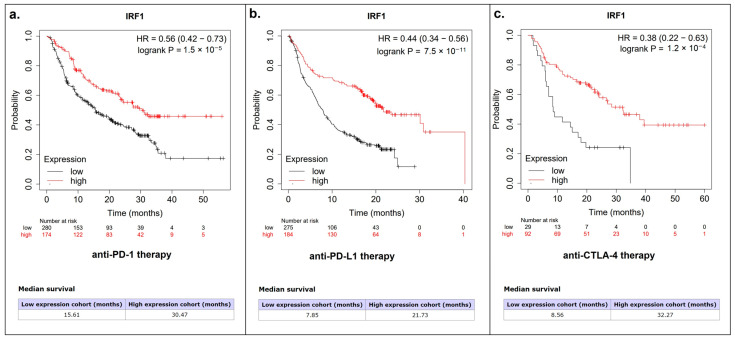
Kaplan–Meier plots based on IRF1 expression in patients receiving (**a**) anti-PD-1, (**b**) anti-PD-L1, and (**c**) anti-CTLA-4 therapy (KMplot database, all tumor types). High IRF1 expression was associated with a more favorable prognosis in all groups.

As a next step, we accessed the pan-cancer mRNA module and immunotherapy module of the KMplot database to investigate the possible association of IRF1 with LUSC patient survival. Although there was no association between IRF1 and overall survival in LUSC patients, regardless of their smoking status, a distinct association with response to anti-PD-(L)1 and anti-CTLA-4 therapy was found (Figure 8a–c).

## 4. Discussion

The connection between smoking and the development of various human diseases and malignant tumors has been widely demonstrated. Tobacco smoke and its constituents are known to affect a number of carcinogenic molecular pathways in LC [29]. Although it is commonly known that tobacco smoke exposure significantly affects the development and progression of LC, research is still ongoing to identify the key mechanisms and their possible practical applications. A variety of smoking-related biomarkers have been investigated, including tobacco smoke toxicants and their metabolites, multiple driver mutations, microRNAs, and others [30,31,32]. In particular, microRNAs have been linked to the regulation of certain oncogenic targets associated with smoking, as well as tumor responses to treatment [33]. In our previous work, we found an association between microRNA-301a and smoking status in patients with LUSC, and this paper aims to further investigate the downstream smoking-related molecular events involving hsa-mir-301a.

MicroRNA-301a has previously been shown to be engaged in several molecular and cellular interactions that contribute to tumor growth. It has been shown to affect signaling pathways (PI3K/AKT, WNT, JAK/STAT, and TGF-β), transcription factors (IRF1, NF-kB, FOXL1, FOSL2, and others), and cancer-related cellular processes like epithelial–mesenchymal transition, cell migration, metabolism, apoptosis, cell cycle progression, hypoxia, and autophagy [34]. MiR-301a has also been previously indicated as a promising potential biomarker for several cancer subtypes, including NSCLC [35,36,37]. Nevertheless, its most important molecular interactions involved in LC development, as well as their potential triggers, are still not fully understood. Here, using bioinformatic research, we not only show increased levels of hsa-mir-301a in patients with a positive smoking status, but also identify its primary smoking-related target—IRF1. Among other putative hsa-mir-301a targets in LUSC, IRF1 mRNA was the only one to meet three key criteria at once: a high target prediction score, a negative correlation with hsa-mir-301a in LUSC, and evidence of an association with patient’s smoking status. IRF1 downregulation, which was found to negatively correlate with hsa-mir-301a upregulation in LUSC, was not only observed in the bioinformatic research but was also confirmed experimentally in clinical LUSC tumor samples. As a result, here we propose IRF1 as a key smoking-related hsa-mir-301a target in LUSC. However, an important limitation of this study should be noted: the absence of a control group of non-smoking patients, which occurred due to the low incidence of LUSC in non-smokers. In future studies, comparing the levels of hsa-mir-301a and IRF1 mRNA between smoking and non-smoking patients may provide more insights into how the identified miR-301a/IRF1 axis depends on the patient’s smoking status.

The IRF1 protein is a transcription factor best known for its involvement in interferon signaling pathways that regulate immune responses. Apart from its initial ability to impact the cellular response to pathogens, its numerous connections to carcinogenesis and the antitumor immune response have also been revealed [38]. Remarkably, IRF1 has already been studied as a target of hsa-mir-301a in hepatocellular carcinoma cells exposed to chronic hypoxia [39]. IRF1′s potential involvement in smoking-induced inflammation in human aortic smooth muscle cells has also been demonstrated [40]. It has not yet been well investigated, however, whether the mir301a/IRF1 axis relates to the carcinogenic pathways driven by tobacco exposure in LUSC or other malignant tumors.

In the present study, we used bioinformatic tools to determine the main downstream molecular pathways affected by IRF1 in LUSC. Bioinformatic data clearly demonstrated the involvement of different immunoregulatory pathways, which was in good agreement with the connection between IRF1 expression levels and tumor immune infiltration in LUSC. Previously, IRF1 was demonstrated to play a role in both activating antitumor immunity and suppressing it through different pathways [41,42]. IRF1 is commonly known for its ability to suppress antitumor immune responses by directly increasing the expression of the *CD274* gene, which encodes PD-L1, an established tumor immune checkpoint molecule [43]. In our study, IRF1 mRNA levels in LUSC were also found to positively correlate with the mRNA levels of all the accessed immune checkpoint molecules currently used for cancer targeted therapy. All the PD-L1, PD-1, CTLA-4, and LAG-3 molecules showed a strong positive correlation with IRF1. High IRF1 expression rates have also been associated with better responses to anti-PD-(L)1 and anti-CTLA-4 immune checkpoint blockade (ICB) therapy, according to our bioinformatic findings. It is important to note that the potential application of IRF1 in predicting response to immunotherapy could be a promising area for further research.

Numerous studies are being conducted to identify novel biomarkers for evaluating responses to ICB immunotherapy [44,45]. A number of significant limitations have been observed in determining the level of PD-L1 in tumor cells, which is currently used as a biomarker for ICB therapy. A lack of response to ICB therapy in PD-L1-positive tumors and responsiveness in PD-L1-negative tumors are both frequently reported, with different explanations proposed for these findings [46,47,48]. In particular, it has been previously reported that LUSC showed no significant differences in response to anti-PD-(L)1 immunotherapy depending on PD-L1 status [46]. In this regard, given the evidence for a close relationship between IRF1 and PD-L1, IRF1 might serve as another possible biomarker to evaluate responses to ICB therapy in LUSC or other malignancies. In fact, IRF1 has previously been reported to be involved in antitumor neutrophil production and the neutrophil interferon response required for tumor control during immunotherapy [49]. Furthermore, nuclear expression of IRF1 has been suggested as a potential biomarker for anti-PD-1 therapy in metastatic melanoma, as a possible means of measuring cells’ ability for PD-L1 expression [50]. Its exact relevance in predicting the response to various immunotherapeutic treatments in LUSC or other cancers, however, has yet to be investigated. The suppression of IFNγ signaling, an important regulator of cellular IRF1 levels, has also been proposed as an important factor contributing to immunotherapy resistance. It is also highlighted that further research is required to determine whether IFNγ can be used in combination with anti-PD-(L)1 therapy to overcome this resistance and increase treatment efficacy [51,52]. Thus, further research on IFNγ signaling in general and IRF1 in particular could provide additional knowledge about ICB treatment response and resistance.

Undoubtedly, additional research is required to fully determine all the molecular interactions underlying such connections and their clinical significance, including the smoking-related mir301a/IRF1 pathway discussed in this study. All upstream interactions leading to the upregulation of hsa-mir-301a also need to be elucidated, as well as the importance of this particular smoking-associated molecular pathway in determining the efficacy of ICB therapies, including anti-PD-(L)1 and others. In total, these findings suggest that the mir301a/IRF1 axis in LUSC or related smoking-associated pathways could be a promising subject for future studies to help clarify the potential clinical benefits of investigating such molecular pathways for evaluating cancer immunotherapy responses.

## 5. Conclusions

In conclusion, the current study allowed us to identify the smoking-related hsa-mir-301a/IRF1 molecular axis in LUSC. Furthermore, bioinformatic data clearly demonstrate the potential connection between IRF1 and different immune pathways in LUSC, as well as the efficacy of immune checkpoint inhibitor therapy. These data could not only clarify some of the molecular characteristics of lung cancer pathogenesis, but also open new perspectives in the future search for beneficial biomarkers for ICB therapy.

## Figures and Tables

**Table 1:** Clinicopathological characteristics of LUSC patients. COPD—chronic obstructive pulmonary disease, CVD—cardiovascular disease.

Characteristics	Number of Patients (%)
Age (years)	
≤65	28 (50)
>65	28 (50)
Gender	
Male	55 (98)
Female	1 (2)
Smoking history	
Current smokers	56 (100)
Pack-years	
≤40	32 (57)
>40	24 (43)
Family history of cancer	
Yes (lung cancer)	8 (14.3)
Yes (other cancer types)	12 (21.4)
No	36 (64.3)
Comorbidities	
COPD	26 (46.4)
CVD without COPD	22 (39.3)
None	8 (14.3)
Lymph node metastasis	
No	34 (60.7)
Yes	22 (39.3)
Pathologic stage (AJCC staging)	
I	22 (39)
II	24 (43)
III	10 (18)

## Data Availability

The data presented in this study are available in the previously listed online databases, reference numbers 20-27. These data were derived from the following resources available in the public domain: UALCAN Database: http://ualcan.path.uab.edu/analysis.html (accessed on 7 May 2024); MirDB Database: https://mirdb.org/ (accessed on 7 May 2024); LinkedOmics Database: http://www.linkedomics.org/ (accessed on 7 May 2024); TargetScan Database: https://www.targetscan.org/vert_80 (accessed on 7 May 2024); Metascape Database: https://metascape.org (accessed on 7 May 2024); Cancerhallmarks Database: https://cancerhallmarks.com/ (accessed on 7 May 2024); TFlink Database: https://tflink.net/ (accessed on 7 May 2024); TIMER Database: https://cistrome.shinyapps.io/timer/ (accessed on 7 May 2024); Mplot Database: https://kmplot.com/analysis/ (accessed on 7 May 2024).

## Supplementary Materials

The following supporting information can be downloaded at: https://www.mdpi.com/article/10.3390/cancers16122208/s1, Table S1: Oligonucleotide sequences used in present study; Table S2: Top hsa-miR-301a-3p targets (miRDB database); Table S3: Genes of top mRNAs negatively correlating with hsa-miR-301a-3p in LUSC (LinkedOmics database); Table S4: Comparison of hsa-miR-301a-3p target lists in LUSC (miRDB, LinkedOmics and UALCAN databases); Table S5: Genes of top mRNAs negatively correlating with IRF1 mRNA in LUSC (LinkedOmics database); Table S6: Targets of IRF1 (TFLink database); Table S7: Targets of IRF1 positively correlating with IRF1 mRNA in LUSC (LinkedOmics and TFLink databases).
